# The effect of transcranial pulse current stimulation on the accumulation of exercise-induced fatigue in college students after moderate intensity exercise evidence from central and peripheral sources

**DOI:** 10.3389/fphys.2025.1502418

**Published:** 2025-02-14

**Authors:** Qingchang Wu, Siyan Liu, Changli Wu, Jian Liu

**Affiliations:** ^1^ College of Physical Education, Soochow University, Suzhou, China; ^2^ College of Physical Education, Nantong University, Nantong, Jiangsu, China; ^3^ Hubei International Travel Health Care Center (Outpatient Department of Wuhan Customs Port), Wuhan, China; ^4^ College of Physical Education, Shenzhen University, Shenzhen, Guangdong, China

**Keywords:** fatigue accumulation, transcranial pulse current stimulation, central fatigue, peripheral fatigue, behavioral indicators

## Abstract

**Objective:**

To investigate the intervention effect of cranial pulse current stimulator (tPCS) on fatigue accumulation after moderate-intensity exercise by using blood analysis and functional near-infrared spectroscopy, and to analyze the type and magnitude of the fatigue effect of tPCS on fatigue in combination with behavioral performance.

**Methods:**

Ninety healthy college students were randomly and equally divided into an experimental group (Group A) and a control group (Group B), and both groups underwent moderate-intensity training for 7 days. Before and after the experiment, all subjects received physiological, biochemical, behavioral, and subjective fatigue indexes, followed by exercise training, and each day of exercise training was followed by tPCS intervention (stimulus intensity of 1.5 mA, stimulus duration of 20 min) and subjective fatigue scale (RPE) test.

**Results:**

① After the tPCS intervention, the daily RPE scores of group A were smaller than those of group B; ② The values of the indexes oxygenated hemoglobin concentration (Oxy-Hb), deoxyhemoglobin concentration (HHb), testosterone (T), and testosterone-to-cortisol ratio (T/C) of group A did not differ significantly from those of the pre-intervention period, and the values of all the indexes of group B were significantly different from those of the pre-intervention period. ③ After tPCS intervention, the values of Oxy-Hb, T, T/C, and on-attention decreased in Groups A and B, with Oxy-Hb decreasing the most; the values of HHb, total hemoglobin concentration (HbTot), hemoglobin concentration difference (HbDiff), cortisol (C), creatine kinase (CK), and reaction time (RT) increased, with the greatest increase in HbDiff; and the Group A The magnitude of change of each index was smaller than that of Group B. After tPCS intervention, the contribution of central fatigue to the effect of reaction time science was greater than that of peripheral fatigue.

**Conclusion:**

① tPCS can delay the development of central fatigue and peripheral fatigue. ② The effect of tPCS on central fatigue is greater than on peripheral fatigue. ③ The effect of tPCS on reaction timing is mainly realized by changing the state of central fatigue.

## 1 Introduction

In sports competitions, fatigue is inevitable, and it can lead to a decline in athletic performance. With the increasing number of sports events, athletes are facing long-term competition challenges, which creates favorable conditions for the accumulation of fatigue. The accumulation of fatigue not only affects the physiological characteristics of individual muscles, such as reducing muscle strength and endurance (Ament and Verkerke, 2009), but also may trigger emotional fluctuations, anxiety, and depression symptoms, which may lead to a decrease in athletes’ motivation and training investment (Pageaux and Lepers, 2018), ultimately inevitably affecting athletes’ competitive performance. Accumulation of fatigue may interfere with neuromuscular control, increase the risk of injury (Jones et al., 2017), and shorten an athlete’s career. Therefore, how to eliminate the accumulation of fatigue and keep athletes in a high-level competitive state is not only of great significance for achieving excellent results, but also has a positive effect on extending the professional life of athletes. Transcranial pulsed current stimulation (tPCS), as a new non-invasive brain stimulation technique, can modulate neuronal activity by delivering oscillatory currents to the cerebral cortex. Compared with transcranial direct current, continuous pulse stimulation of tPCS can cause repeated depolarization of cells, resulting in a cumulative effect of neural excitation and ultimately leading to greater cortical excitability changes (Datta et al., 2013; Ma et al., 2019). Due to its unique bipolar pulse characteristics, some scholars have used it in the fields of eliminating exercise fatigue and exercise cognition (Morales-Quezada et al., 2015; Wu et al., 2022). Therefore, in this study, tPCS was used as an intervention to combat fatigue accumulation. Previous studies have shown that most scholars have studied the immediate effects of tPCS intervention once, and have not yet paid attention to the cumulative effects of interventions on tPCS. Moreover, the occurrence of fatigue has a cumulative effect, and the Rating of Perceived Exercise (RPE) is more sensitive to measuring fatigue accumulation (Martin and Andersen, 2000). Therefore, studying the effect of tPCS on fatigue accumulation under long-term exercise can improve the application program of tPCS and expand its application scenarios, which has a positive significance for maintaining athletic performance (Kataoka et al., 2022).

According to the location of fatigue, it can be divided into central fatigue and peripheral fatigue; Its monitoring methods can be divided into behavioral, biochemical, and physiological categories, which measure the degree of fatigue from subjective and objective dimensions, respectively. The monitoring of behavioral indicators of fatigue refers to the completion of specific actions by subjects according to established procedures (Li and Zhang, 2015; Qin, 2006), and the generation of actions is influenced by central fatigue - relying on neural activation to transmit signals; It is also affected by peripheral fatigue - relying on favorable external conditions of muscle tissue, such as sufficient energy supply or timely clearance of metabolites (Amann, 2011). This means that the completion of actions is influenced by both central fatigue and peripheral fatigue, therefore, the study analyzed the elimination effect of tPCS intervention on fatigue accumulation from both central and peripheral perspectives. Biochemical indicators are powerful evidence of peripheral fatigue (Qiu, 2022; Antunes et al., 2016), while testosterone (C), cortisol (T), and creatine kinase (CK) are often used in fatigue testing, and T/C is widely recognized as a sensitive indicator of fatigue status (Freitas et al., 2014; Selmi et al., 2022). Functional near-infrared spectroscopy (fNIRS) is a physiological fatigue monitoring method that uses non-invasive brain imaging technology to dynamically monitor brain blood oxygen signals to reflect the activation status of brain nerves. Insufficient oxygen delivery and/or low arterial oxygen pressure gradient in the brain can affect the diffusion of oxygen to the sarcomere and mitochondria, leading to central fatigue, FNIRS monitoring of cerebral blood oxygen can reflect central fatigue status (Amann and Calbet, 2008; Liu Jianxiu et al., 2016). Indeed, previous studies have shown a relationship between NIRS-related measures and measures of central fatigue (Ruggiero and McNeil, 2019). In behavioral indicators, reaction time and attention are commonly used to reflect fatigue status, and when fatigue occurs, reaction time becomes longer or attention decreases (Harrison et al., 2017). Previous studies have rarely analyzed the effects of tPCS on fatigue from three different fatigue monitoring perspectives, and the behavioral indicators are influenced by central fatigue or (and) peripheral fatigue (Liu T. et al., 2016; Carroll et al., 2017; Drummond et al., 2005). Therefore, we speculate that the changes in behavioral indicators induced by tPCS may be caused by peripheral fatigue or (and) central fatigue.

In summary, this study aims to explore the intervention effect of tPCS on fatigue accumulation after 7 days of moderate intensity training; And analyze from the perspective of fatigue generation mechanism which type of fatigue elimination effect tPCS has better, to guide the application of tPCS in practice; Simultaneously explore the fatigue pathway through which tPCS intervention promotes changes in behavioral indicators. Based on this, this study assumes that ① tPCS has a cumulative effect on the elimination of fatigue. ② tPCS has an eliminating effect on both types of fatigue, with a greater effect on central fatigue ③ The changes in behavioral indicators induced by tPCS are jointly caused by central fatigue and peripheral fatigue (Weng et al., 2016).

## 2 Research object and methods

### 2.1 Ethics

All of the participants signed the informed consent form. The study was carried out in line with the Declaration of Helsinki and was approved by the Institutional Ethics Committee (16 November 2020 (no. 1) by Nantong University).

### 2.2 Research object

**FIGURE 1:**
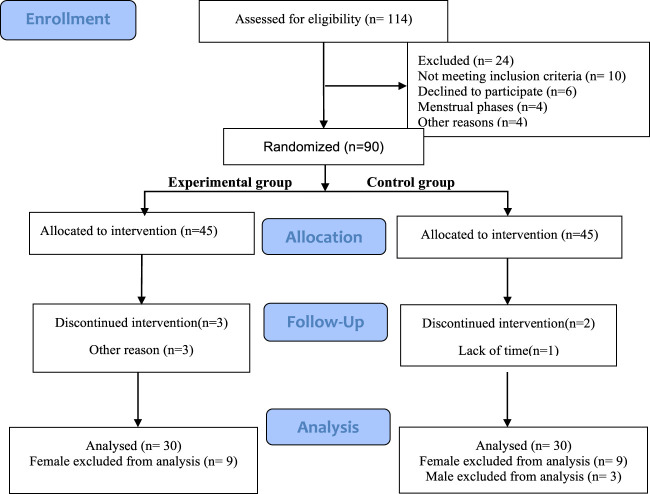
Inclusion subject flowchart.

This study selected 90 healthy college athletes as participants, using a simple randomized scale method. Odd numbers were used as the experimental group, and even numbers were used as the control group. After multiple allocations, each group had 45 participants. After screening, 30 participants (all participants are male) from each group participated in the entire experimental process (Figure 1). The average age of the true stimulation group was 20.47 ± 0.72 years, with a training period of 4.12 ± 0.99 years and an average height of 177.89 ± 7.24 cm, The average weight is 73.97 ± 10.99 kg; The average age of the sham stimulation group was 20.63 ± 1.46 years, with a training period of 4.00 ± 0.75 years, an average height of 178.37 ± 6.40 cm, and an average weight of 70.58 ± 8.96 kg. All voluntary athletes participating in this study are ordinary college athletes with good physical condition and long-term running experience, without cardiovascular diseases or other illnesses, and have no injuries in the first 4 weeks. They did not engage in physical exercise in the week before the experiment and are all right-handed. During the experiment, the athletes did not participate in any activities that could lead to a decrease in physical fatigue (such as massage, or physical therapy) or an increase (such as overtraining). Before the test, participants received explanations about transcranial pulse electrical stimulation, experimental objectives, and experimental procedures.

The sample size was calculated by G*Power 3.1. the ANOVA test method was selected, According to previous studies (Joundi et al., 2012; Pollok et al., 2015), the effect size of o.6. With α error probability of 0.05 and power (1-β error probability) of 0.8, the resulting sample size was 20. Considering potential dropouts, we recruited a bit more participants.

### 2.3 Experimental process

The 90 athletes in this study were randomly divided into two groups, Group A being the experimental group and Group B being the control group. All participants completed this study in two stages: the familiarization stage and the formal experiment stage. The familiarization stage requires participants to be familiar with all testing tasks, intervention processes, and indicator testing processes to reduce the impact of learning effects and physical discomfort on formal experiments.

The content of the familiarization section includes.

1) Familiarize oneself with sports training plans and grasp the key points during training;
2) Familiar with the instruments and testing indicators used in this research institute;
3) Finally, inform the subjects of precautions.

**FIGURE 2:**
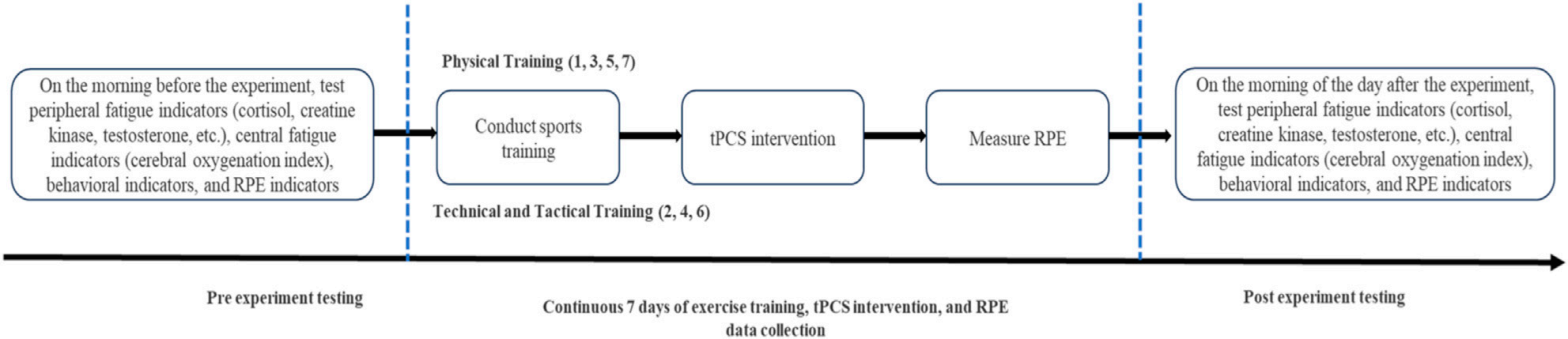
Formal experimental flowchart.

The flowchart of the formal experiment (Figure 2) shows that tPCS intervention is performed immediately after completing exercise training every day, and RPE is measured after the intervention is completed.

### 2.4 Exercise plan

To fit the actual sports scenario, physical training and tactical training were alternated, and all participants used the moderate intensity training standard recommended by the American College of Sports Medicine (heart rate 140–150 beats per minute (ACSM., 2013). The athletes conducted a 1-week (7 days) training. The physical training tasks are conducted on the first day, third day, fifth day, and seventh day, the main training is for long-distance running. On the second, fourth day, and sixth days, technical and tactical training tasks were carried out, mainly for special technical action training. Group A and Group B are required to participate in training every day, with a training duration of approximately 3 h per day. During the main training period, the intensity was maintained at a heart rate of 140–150 beats per minute, and the RPE score exceeded eight points after training (Table 1).

**TABLE 1:** Training volume and training time.

Day	Total training distance/km	Main training Time/min	Auxiliary training Time/min	Borg
First day	20 km	138 min	42 min	8.53 ± 0.67
Second day	14 km	108 min	72 min	8.26 ± 0.44
Third day	22 km	144 min	36 min	8.80 ± 0.75
Fourth day	16 km	113 min	67 min	8.36 ± 0.60
Fifth day	18 km	124 min	56 min	9.00 ± 0.68
Sixth day	14 km	110 min	70 min	8.66 ± 0.65
Seventh day	18 km	130 min	50 min	8.93 ± 0.72

Note: Main training refers to formal running time and auxiliary training refers to technical movement preparation, simulation training, warm-up, and so on.

The specific training plan is as follows:

Before training, the subjects are familiar with the RPE scale. The exercise trainer supervises the training and evaluates the exercise intensity of the subjects through the RPE scale and heart rate.

**FIGURE 3:**
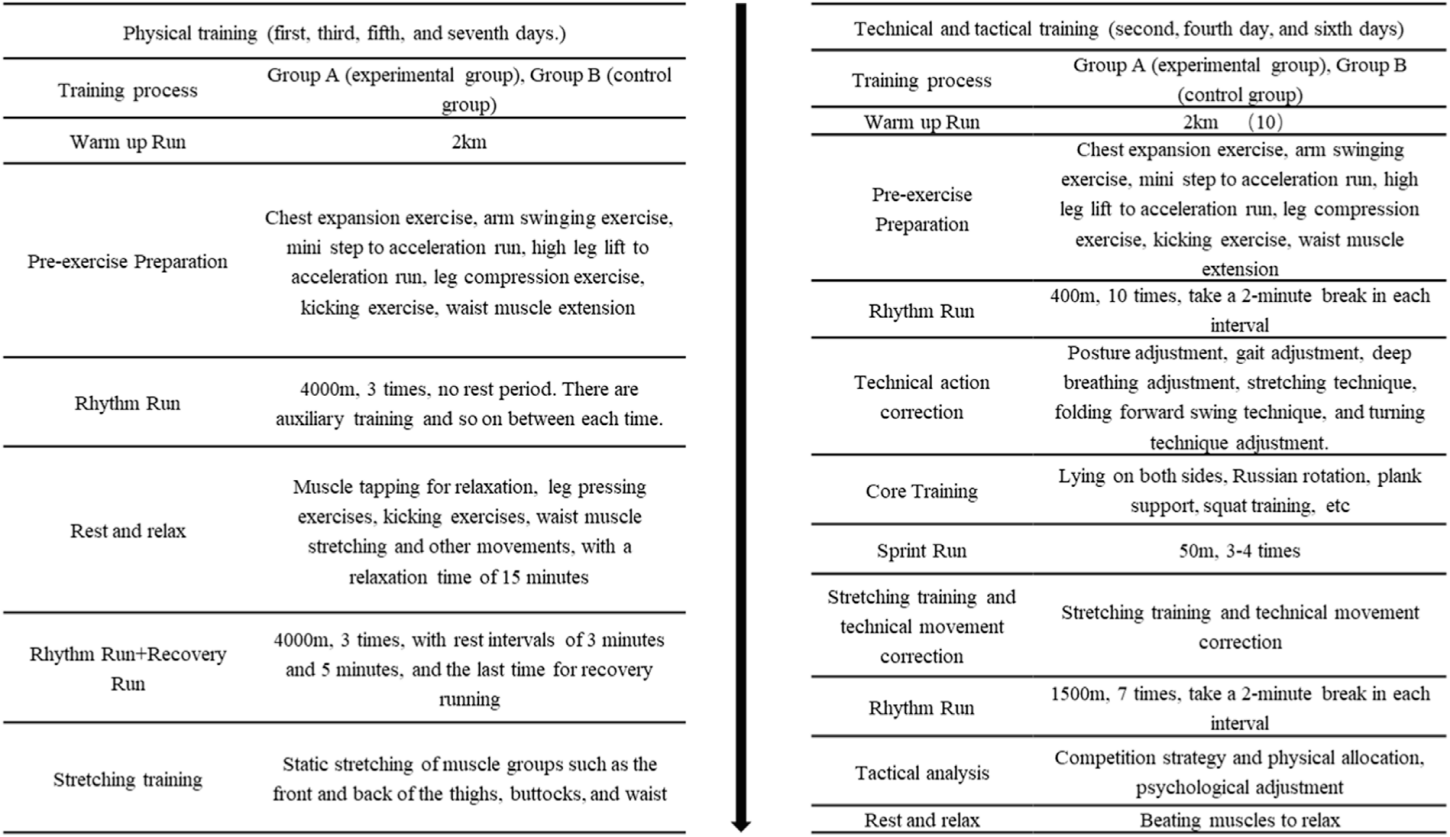
Formal training content chart.

Every afternoon at 3 o’clock, the subjects start training. All participants wore Firstbeat Sports to monitor heart rate and collect RPE baseline before training. Subsequently, both Group A and Group B participants received warm-up runs and Pre-exercises preparation before starting formal training. The training content of Group A and Group B is shown in Figure 3.

### 2.5 Transcranial pulse current intervention plan

This study used a transcranial pulse current stimulation independently developed by the project team for intervention. To ensure the safety of all subjects, the intervention plan was based on the study by Dissanayaka T et al. (2020), with a stimulation intensity of 1.5 mA and a stimulation time of 20 min. Firstly, place a rectangular electrode piece with a size of (5 × 9) cm^2^ in the center of the forehead, and place two rectangular electrode pieces with a size of (5 × 5) cm^2^ at the bilateral papillae. Subsequently, increase the current intensity to 1.5 mA within 30 s and continue for 20 min; During the stimulation process, participants are required to maintain an upright and static sitting position to avoid external interference; After the stimulation ends, adjust the current intensity to 0 mA within 30 s. All conditions for false stimulation are the same as true stimulation, except that after the initial acceleration reaches 1.5 mA, the operator readjusts the current to 0 mA. All conditions for false stimulation are the same as true stimulation, except that the operator readjusts the current to 0 mA after the initial acceleration reaches the target value. All operations were completed by the same personnel, and any discomfort experienced by the subjects during the operation should be promptly reported to the experimenters.

### 2.6 Experimental equipment

#### 2.6.1 Transcranial pulse current stimulation (tPCS)

Developed by the National Key Technology R&D Program of China, the stimulation current is a bipolar current of 60–80 Hz, the pulse waveform is a square wave, the duty cycle is 29.7%, the stimulation intensity is in the range of 0–2 mA, and the stimulation time is determined by the experimental program. This product passed national security certification on 17 April 2021: report number CHTSM21040049.

#### 2.6.2 Blood collection

We used 5 mL EDTA-anticoagulated vacutainer tubes to collect blood samples from the participants, which were produced by Lingen Precision Medical Products (Shanghai) Co., Ltd. (Shanghai, China).

#### 2.6.3 Functional near-infrared spectroscopy

**FIGURE 4:**
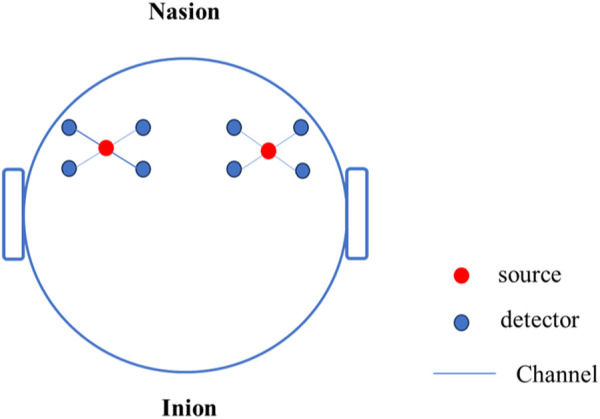
Functional near-infrared layout diagram.

This study used an OctaMon + portable wireless near-infrared brain imaging system (Artinis, Netherlands) to collect Oxy Hb concentration data in the frontal lobe of the brain. OctaMon has a total of eight light source emission stages, generating 760 nm and 850 nm light waves, two light source detectors, and a sampling frequency of 50 Hz. According to the international 10–20 system brain electrode standard lead, the fNIRS channel position was registered with MNI spatial coordinates using a 3D locator and probability registration method. The fNIRS detector covered the frontal eye fields (FEF), dorsolateral prefrontal cortex (DLPFC), and orbitofrontal cortex (OFC), as shown in Figure 4.

#### 2.6.4 Behavioral testing instruments

We use instruments produced by the Science and Education Instrument Factory of East China Normal University. The model of the reaction time tester is EP202.203, and the model of the attention concentration tester is EP701C.

#### 2.6.5 RPE questionnaire

Using the RPE 10-level scale, the higher the RPE score, the greater the level of fatigue (Zhu et al., 2020).

### 2.7 Testing indicators

All data collection was conducted using a sitting position test. The physiological indicators of oxygenated hemoglobin (Oxy-Hb) concentration, deoxyhemoglobin (HHb), total hemoglobin (HbTot) concentration, and hemoglobin difference (HbDiff) were measured by the OctaMon + portable wireless near-infrared brain imaging system; Biochemical indicators such as testosterone (T), cortisol (C), testosterone/cortisol (T/C), and creatine kinase (CK) were measured by a fully automated biochemical analyzer (RaytoRT-6100 China); The reaction time (RT) of behavioral indicators was measured by the EP202.203 reaction time tester produced by the Science and Education Instrument Factory of East China Normal University, and attention was measured by the EP701C attention concentration tester of East China Normal University.

### 2.8 Data collection

#### 2.8.1 Collection of physiological indicators

First, nirsLAB v. 2013.1 software (Version 14, Revision 2, NIRx Medizintechnik GmbH, Berlin, Germany) was used to convert the collected original data into MATLAB format data. Then, the differential function DIFF in MATLAB was used to observe whether there was a horizontal signal in the signal. If there were more than 25% invalid horizontal signals in the signal, we excluded the signal of this channel. Then, Butterworth filtering was used to reduce the interference of high-frequency noise (0.3 Hz respiration and 1 Hz heart rate) and low-frequency noise (metabolic tremor of less than 0.01 Hz) in the signal and improve the signal-to-noise ratio (BORG, 1988). Using the principal component analysis method proposed by Yücel (Yücel et al., 2014) to remove movement artifacts, the calculated monitoring time of the oxygenated hemoglobin concentration was 3 min. Finally, the oxygenated hemoglobin concentration data of all subjects were calculated, and the change in the prefrontal lobe oxygenated hemoglobin concentration (Oxy-Hb) was calculated according to the improved Beer–Brown law. Collection of Biochemical Indicators.

#### 2.8.2 Collection of Biochemical Indicators

In this experiment, blood was collected twice, the day before and the day after the experiment. After the subjects reached the blood collection room (the temperature is 20°C), they sat quietly and rested for 10 min, and then the medical staff took 4 mL of left elbow venous blood and collected the blood sample in a vacuum blank tube to avoid shaking and vibration of the contents (blood). Within 30 min, serum was separated using a high-speed centrifuge (2000 R/min, 15 min. Shu Ke, China). The supernatant was extracted and stored in a medical refrigerator at −80°C (Boko, BDF-86V158, China). CK, T, and C were detected using ELISA kits (Wuhan Jianglai Biotechnology Co., Ltd. (Wuhan, China), according to the manufacturer’s instructions, and the whole process was supervised by a principal investigator. All samples were sent to the laboratory for biochemical analysis within 48 h, and all analyses were repeated and performed by trained technicians. Serum samples were analyzed using an automatic biochemical analyzer (RaytoRT-6100 China). Serum CK, T, and C levels were recorded, and the serum T/C ratio was calculated.

#### 2.8.3 Collection of behavioral indicators

The RT tester (EP202.203) produced by Shanghai East China Normal University Science and Education Instrument Co., Ltd. was applied to compare the choice RT of the subjects under light stimulation of four colors (red, green, yellow, and blue). By the way, a four-hole photoelectric contactless response key acted as the response component of the subjects, with 20 stimuli per test (5 times for each color). At the beginning of the test, the instrument automatically and randomly presented the four-color light stimulus. According to the presentation of the light stimulus, the fingers of the subjects left the middle position of the response keyboard and pressed the circular hole of the corresponding color. The instrument automatically recorded the time between stimulus presentation and the fingers of subjects entering the circular hole of the corresponding response keyboard, and the mismatched color that subjects pressed would automatically be processed as an error by the instrument, and the corresponding time was not counted in the statistics. After completion of 20 tests, the buzzer in the instrument automatically sounded for 1 s for a hint. Finally, the total average RT was recorded (Deng et al., 2013).

An attention concentration tester (EP701C) produced by Shanghai East China Normal University Science and Education Instrument Co., Ltd. was adopted for the examination of the attention concentration ability of individuals. To be specific, the instrument was placed on a table about 1.30 m high, and then the subjects held the induction handle with a handedness to trace the white sign of rotation in the hexagonal track. Before measurement, the instrument was adjusted in advance, the movement speed of the white mark was set as 30 r/min, and the test time as 20 s. The instrument automatically recorded the onattention and off-attention after completion of the test. The test rules were explained first, then the subjects were allowed to practice once and measure once (Li et al., 2017).

### 2.9 Statistical analysis

All data were analyzed using SPSS 25.0 and graphically plotted using Graph Pad Prism 8.0 software. Firstly, perform a normal distribution test on the data, and use Mann Whitney U test for data that does not conform to a normal distribution. Using stimulus conditions (Group A, Group B) as inter-group variables and time (pre-test, post-test) as intra-group variables, a two factor repeated measures analysis of variance was conducted to investigate the effects of each variable on physiological, biochemical, and behavioral indicators. The effect size was represented by a bias of η^2^. Use independent sample t-test to analyze the RPE differences between the two groups. Pearson bilateral correlation test was used to analyze the correlation between various indicators, and linear regression was used to explore the contributions of central fatigue and peripheral fatigue to behavioral changes. The data for statistical testing is expressed as (M ± SD), with a 95% confidence interval. P < 0.05 indicates significant differences, while P < 0.01 indicates very significant differences.

## 3 Results

### 3.1 Daily RPE score after training

**FIGURE 5:**
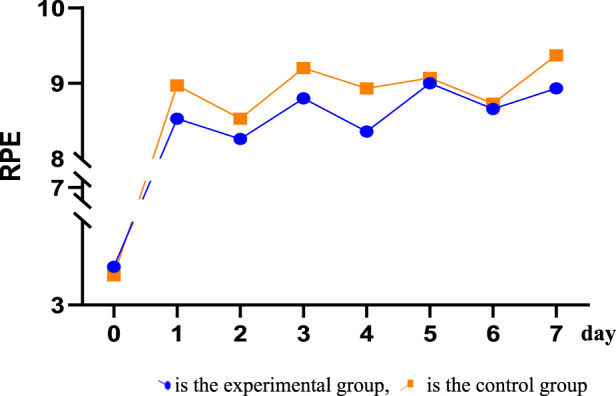
Trend of RPE scores during training.

After receiving tPCS intervention, all subjects underwent RPE score recording. The results showed that the daily RPE score exceeded eight points, and the RPE score after physical training was higher than 8.5 points, while the RPE score after technical and tactical training was less than 8.5 points. The independent sample T-test was used to analyze the RPE values of the two groups. The results showed that there were significant differences in RPE scores between the two groups on the first day, the third day, the fourth day, and the seventh day. The RPE scores of the control group were higher than those of the experimental group (Figure 5; Table 2).

**TABLE 2:** RPE scoring checklist.

Group	Baseline	First day	Second day	Third day	Fourth day	Fifth day	Sixth day	Seventh day
Experimental group (A)	4.07	8.53	8.26	8.80	8.36	9.00	8.66	8.93
Control group (B)	3.83	8.97	8.53	9.20	8.93	9.07	8.73	9.37
t	0.40	−2.70	−1.70	−2.10	−3.23	−0.36	−0.38	−2.47
*p*	0.69	0.01	0.09	0.04	0.00	0.72	0.70	0.02
RPE difference	−0.24	0.44	0.27	0.40	0.57	0.07	0.07	0.44

### 3.2 Baseline data results before tPCS intervention

After completing exercise training, all participants measured their baseline values and conducted independent sample t-test analysis. It was found that there was no significant difference between Group A and Group B in physiological, biochemical, and subjective indicators (Table 3).

**TABLE 3:** Test results of various indicators before tPCS intervention.

Indicators	Group A	Group B	t	p
	M ± SD	M ± SD		
Oxy-Hb	−6.88 ± 8.189	−7.50 ± 7.23	0.31	0.76
HHb	4.06 ± 2.73	4.46 ± 2.24	−0.62	0.54
HbTot	12.77 ± 9.74	12.91 ± 8.34	−0.06	0.95
HbDiff	3.37 ± 5.19	3.62 ± 5.01	−0.19	0.85
T (nmol/L)	5.68 ± 0.71	5.57 ± 0.59	0.61	0.55
C (nmol/L)	255.11 ± 31.10	245.70 ± 18.05	1.43	0.16
T/C	0.07 ± 0.01	0.07 ± 0.00	−1.67	0.10
CK(ng/mL)	80.51 ± 10.38	79.11 ± 11.75	0.49	0.63
RT	0.37 ± 0.10	0.37 ± 0.13	−0.23	0.82
attention	14.14 ± 1.64	14.01 ± 1.46	−0.32	0.75

### 3.3 Changes in various indicators after tPCS intervention

#### 3.3.1 Physiological indicators

After completing the training, participants underwent tPCS intervention. Two-way repeated measures ANOVA showed significant main effects for time on Oxy-Hb [F (1,58) = 10.95, p = 0.00, η^2^ = 0.16], HHb [F (1,58) = 4.89, p = 0.03, η^2^ = 0.08], HbTot [F (1,58) = 14.54, p < 0.01, η^2^ = 0.20], and HbDiff [F (1,58) = 8.89, p < 0.01, η^2^ = 0.13].

Post-hoc tests revealed that the experimental group showed a decrease in Oxy-Hb after intervention, with no significant differences compared to pre-intervention or the control group post-intervention. In contrast, the control group showed a significant decrease in Oxy-Hb after intervention compared to pre-intervention (p < 0.01). The experimental group also exhibited increases in HHb, HbTot, and HbDiff post-intervention, with no significant differences compared to pre-intervention or the control group. The control group showed significant increases in HHb, HbTot, and HbDiff post-intervention compared to pre-intervention (p < 0.01) (see Table 5).

#### 3.3.2 Biochemical indicators

After completing the training, participants underwent tPCS intervention. Two-way repeated measures ANOVA revealed significant interaction effects for cortisol, T/C ratio, and CK across stimulation conditions and time, specifically C [F (1,58) = 14.48, p < 0.01, η^2^ = 0.20], T/C [F (1,58) = 16.40, p < 0.01, η^2^ = 0.22], and CK [F (1,58) = 5.17, p = 0.03, η^2^ = 0.08]. Significant main effects of time were observed for T [F (1,58) = 9.33, p < 0.01, η^2^ = 0.14], C [F (1,58) = 97.97, p < 0.01, η^2^ = 0.63], T/C [F (1,58) = 15.68, p < 0.01, η^2^ = 0.21], and CK [F (1,58) = 90.63, p < 0.01, η^2^ = 0.61]. The main effect for stimulation condition was also significant for C [F (1,58) = 4.14, p = 0.05, η^2^ = 0.07].

Post-hoc tests indicated that the experimental group showed no significant differences in T and T/C after intervention compared to pre-intervention, but significant differences were noted compared to the control group (p < 0.05). The control group showed significant decreases in T and T/C after intervention (p < 0.01). Conversely, the experimental group exhibited significant increases in C and CK after intervention compared to pre-intervention and to the control group (p < 0.01), while the control group also showed significant increases in C and CK post-intervention (p < 0.01) (Tables 5, 6) (p < 0.01) (Tables 4, 5).

**TABLE 4:** Test results of various indicators after tPCS intervention.

Indicators	Group A	Group B	t	p
	M ± SD	M ± SD		
Oxy-Hb	−10.90 ± 8.95	−13.28 ± 10.08	0.97	0.34
HHb	5.07 ± 5.50	6.86 ± 5.75	−1.24	0.22
HbTot	18.30 ± 11.10	20.98 ± 13.43	−0.84	0.40
HbDiff	6.29 ± 0.66	6.82 ± 7.60	−0.29	0.78
T (nmol/L)	5.49 ± 0.71	5.15 ± 0.55	2.16	0.04
C (nmol/L)	287.02 ± 27.15	317.46 ± 34.94	−3.77	0.00
T/C	0.06 ± 0.01	0.05 ± 0.00	3.62	0.00
CK(ng/mL)	95.04 ± 10.81	102.77 ± 11.39	−2.69	0.01
RT	0.41 ± 0.06	0.48 ± 0.103	−0.07	0.00
attention	12.33 ± 1.96	11.12 ± 2.177	1.21	0.03

**TABLE 5:** Test results of various indicators before and after tPCS intervention.

Indicators	Group	T	p	Chang rate	Change rate difference
Oxy-Hb	A	4.01	0.06	58.43%	18.64%
	B	5.78	0.00	77.07%	
HHb	A	−1.09	0.36	24.88%	28.93%
	B	−2.40	0.03	53.81%	
HbTot	A	−5.52	0.03	43.30%	19.21%
	B	−8.07	0.00	62.51%	
HbDiff	A	−2.92	0.05	86.65%	1.75%
	B	−3.21	0.03	88.40%	
T (nmol/L)	A	0.17	0.20	−3.35%	4.20%
	B	0.41	0.00	−7.54%	
C (nmol/L)	A	−31.91	0.00	12.51%	16.70%
	B	−71.76	0.00	29.21%	
T/C	A	0.00	0.95	−12.01%	14.94%
	B	0.00	0.00	−26.95%	
CK(ng/mL)	A	−14.53	0.00	18.05%	11.86%
	B	−23.53	0.00	29.91%	
RT	A	0.04	0.02	10.81%	18.92%
	B	−0.11	0.02	29.73%	
attention	A	1.81	0.00	−12.80%	7.83%
	B	2.89	0.00	−20.63%	

Note: The calculation method for the difference in rate of change R = *|* $\frac{B_{1}-B_{2}}{B_{2}}-\frac{A_{1}-A_{2}}{A_{2}}$ *|*; A1 = numerical value after intervention in Group A; A2 = pre intervention value in Group A; B1 = value after intervention in Group B; B2 = pre intervention value for Group B.

#### 3.3.3 Behavioral indicators

After completing the training, participants underwent tPCS intervention. Two-way repeated measures ANOVA revealed a significant interaction effect of RT across stimulation conditions and time [F (1,58) = 5.32, p = 0.03, η^2^ = 0.08]. There were significant main effects for time on RT [F (1,58) = 25.18, p = 0.00, η^2^ = 0.30] and on target time [F (1,58) = 43.36, p < 0.01, η^2^ = 0.44]. The main effect of the stimulation condition was significant for target time as well [F (1,58) = 4.36, p = 0.04, η^2^ = 0.07].

Post-hoc tests indicated that the experimental group showed a significant increase in RT after the intervention compared to before and the control group. The control group also had a significant increase in RT post-intervention compared to pre-intervention. Conversely, the experimental group experienced a significant decrease in target time after the intervention compared to before and to the control group, while the control group also showed a significant decrease in target time post-intervention compared to pre-intervention (P < 0.01) (Tables 4, 5).

### 3.4 Correlation analysis of various indicators after tPCS intervention

Pearson correlation analysis revealed significant relationships between physiological, and biochemical indicators, and reaction time following tPCS intervention. In Group A, reaction time was significantly negatively correlated with T, T/C, and Oxy-Hb, and positively correlated with HHb, HbTot, and HbDiff. T was significantly positively correlated with T/C and Oxy-Hb, and negatively correlated with HHb, HbTot, and HbDiff. In Group B, reaction time was significantly negatively correlated with T, T/C, and Oxy-Hb, and positively correlated with HbDiff. T was significantly positively correlated with T/C and Oxy-Hb, and negatively correlated with HHb (Table 6).

**TABLE 6:** Correlation analysis of various indicators after tPCS intervention.

Group	Indicators	RT	Attention	T	C	T/C	CK	Oxy-Hb	HHb	HbTot	HbDiff
A	RT	1	0.02	−0.88**	0.03	−0.64**	−0.20	−0.96**	0.48**	0.46*	0.43*
	attention		1	−0.02	−0.13	0.07	0.015	−0.04	−0.17	−0.14	−0.08
	T			1	0.07	0.66^**^	0.09	0.88^**^	−0.54^**^	−0.48^**^	−0.42^*^
	C				1	−0.69^**^	0.12	−0.09	−0.08	−0.09	−0.01
	TC					1	−0.01	0.67^**^	−0.34	−0.28	−0.28
	CK						1	0.13	−0.07	−0.07	0.04
	Oxy-Hb							1	−0.55^**^	−0.48^**^	−0.39^*^
	HHb								1	0.83^**^	0.57^**^
	HbTot									1	0.88^**^
	HbDiff										1
B	RT	1	0.05	−0.58**	−0.32	−0.62**	0.29	−0.87**	0.05	−0.19	−0.39*
	attention		1	−0.09	0.22	−0.10	0.25	0.01	−0.06	−0.09	0.00
	T			1	0.24	0.96**	−0.32	0.86**	−0.38*	−0.33	0.10
	C				1	0.23	−0.07	0.35	0.02	−0.10	−0.08
	TC					1	−0.24	0.89^**^	−0.36	−0.22	0.17
	CK						1	−0.30	0.04	0.11	0.09
	Oxy-Hb							1	−0.25	−0.06	0.30
	HHb								1	0.52^**^	0.41^*^
	HbTot									1	0.054^**^
	HbDiff										1

Note: **P < 0.01,*P < 0.05.

### 3.5 Regression analysis of central fatigue and peripheral fatigue on response time

A linear regression analysis was conducted with central and peripheral fatigue as independent variables and reaction time as the dependent variable. The results indicated a significant negative effect of central fatigue on reaction time in both groups A and B following tPCS intervention, with group A contributing 92.7% to the effect and group B contributing 86.1% (Table 7).

**TABLE 7:** Regression analysis of central and peripheral fatigue indicators on response time after tPCS intervention.

Group	Indicators	RT			
		SE	Β	t	P
A	T	0.13	0.21	0.60	0.56
	C	5.68	−0.39	−1.19	0.25
	T/C	0.00	−0.46	−1.10	0.28
	CK	1.90	−0.08	−1.45	0.16
	Oxy-Hb	1.50	−0.90	−7.00	0.00
	HHb	0.50	−0.10	−0.79	0.44
	HbTot	1.78	−0.12	−0.57	0.57
	HbDiff	0.95	0.20	1.35	0.19
	*R* ^*2*^	0.93			
	*F*	47.19			
B	T	0.11	0.07	0.24	0.82
	C	3.30	0.04	0.54	0.59
	T/C	0.00	0.60	1.91	0.07
	CK	2.14	0.04	0.46	0.65
	Oxy-Hb	1.32	−1.49	−7.98	0.00
	HHb	0.41	−0.02	−0.22	0.83
	HbTot	1.52	−0.12	−1.15	0.27
	HbDiff	0.92	0.01	0.12	0.90
	*R* ^*2*^	0.86			
	*F*	23.43			

## 4 Discussion

This study investigates the intervention effects of tPCS on exercise-induced fatigue accumulation, and analyze which type of fatigue has a greater intervention effect from both central and peripheral perspectives. The results indicate that the fatigue levels of both groups increased after training, with significant differences in RPE scores compared to baseline. Following tPCS intervention, the experimental group exhibited lower RPE scores than the control group, but with no significant differences between the two groups. This suggests that while natural recovery can delay the rise in fatigue, tPCS intervention is more effective in delaying this increase, supporting Hypothesis 1. Analysis of oxygenated hemoglobin and routine biochemical markers revealed that tPCS impacts both central fatigue and peripheral fatigue, with a stronger effect on central fatigue, confirming Hypothesis 2. Regression results further indicate that the impact of tPCS on exercise behavior is primarily mediated through its effects on central fatigue.

### 4.1 Central evidence of the effect of tPCS on fatigue

The results of fNIRS showed that after daily exercise, the physiological indicators Oxy Hb, HHb, HbTot, and HbDiff in the experimental group were lower than those in the control group. Among them, Oxy Hb, HHb, and HbDiff showed no significant changes compared to before the intervention. This indicates that using tPCS intervention after continuous exercise can maintain the blood oxygen status in the brain and delay the deepening of central fatigue.

In fact, the high or low oxygen content in the brain has been regarded as an important mechanism for the generation and prevention of central fatigue (Nybo and Rasmussen, 2007), and changes in cerebral blood oxygen reflect the activation status of the brain and the degree of central fatigue (Rasmussen et al., 2007). During brain activation, the excitability of neural activity increases, and local brain tissue blood flow, blood volume, and blood oxygen consumption all increase, but the proportion of increase varies. Oxygen consumption only slightly increases, and the increase in blood flow exceeds the increase in oxygen consumption, this difference leads to an increase in oxy-Hb concentration and a decrease in HHb concentration in the brain activation functional area (Hoshi, 2003). This is inconsistent with the results of this study, mainly because the fatigue of the subjects in this study has not been completely eliminated, and fatigue has accumulated after exercise. Therefore, the increase in oxygen consumption exceeds the increase in blood oxygen flow, manifested as a decrease in oxy-Hb concentration and an increase in HHb concentration in indicators; After tPCS intervention, although the concentration of oxy-Hb decreased and HHb increased, the difference in physiological changes in the experimental group was smaller than that in the control group, indicating that tPCS can delay the deepening of central fatigue.

Saavedra et al.’s study showed that tPCS stimulation in the frontal lobe can improve the excitability of the cerebral cortex, strengthen the connections between brain regions, and improve the blood oxygen transport capacity of the stimulated region (Saavedra et al., 2014), which to some extent explains the results of this study. In this study, both groups of subjects showed a decrease in blood oxygen concentration after exercise, but the group treated with tPCS intervention had a lower degree of decrease and no significant change compared to baseline. The reason may be that the subjects of this study received continuous 7 days of exercise training, and the physiological activation of the brain increased with the increase of exercise. Strong neuronal activity caused the demand for blood oxygen to exceed the transport of blood oxygen (Ding, 2012), leading to central fatigue. As time accumulated, the degree of fatigue increased, and the effect of tPCS on increasing the concentration of oxygenated hemoglobin was limited, Interventions that increase cerebral blood flow supply to maintain brain function can only alleviate central fatigue and cannot achieve the effect of eliminating central fatigue. The study by Inglese M et al. also supports this result. Inglese M found through his study of cerebral perfusion that as fatigue increases, cerebral blood flow decreases, and increasing blood flow supply can alleviate fatigue (Inglese et al., 2007).

### 4.2 Peripheral evidence of the effect of tPCS on fatigue

After 7 days of moderate-intensity training, the T and T/C values in the biochemical indicators of the two groups of subjects decreased, while the C and CK values increased, and peripheral fatigue significantly increased. The change rate of group indicators after tPCS intervention was lower than that of the control group, indicating that using tPCS intervention after moderate-intensity training can significantly delay the accumulation of peripheral fatigue. Ma and Gao et al. (2021) found in their study that during continuous high-volume training, the concentrations of CK and C increased over time, while T and T/C showed a significant decrease, which is consistent with the findings of this study. This study adopted a combination of moderate-intensity physical training and technical tactics and found that within a week, the T and T/C values of both groups of subjects were decreasing, while the C and CK values were increasing. This means that after 7 days of training, the physical functions of the subjects decreased and fatigue levels deepened. However, after intervention with tPCS, the changes in biochemical indicators in the experimental group were smaller than those in the control group, indicating that tPCS has the effect of delaying the deepening of fatigue. As Ma and Gao et al. (2021) continued their training, it was found that C and CK values showed a wave-like decrease, while T and T/C showed an increasing trend. However, further research is needed to determine whether continuous tPCS intervention can further delay fatigue and even improve subject status. There are also scholars whose research results are different from this study. Ma Haihao’s research shows that after a week of high-altitude training, the T and T/C values in athletes’ blood significantly increase compared to a week ago, while the C value first increases and then decreases. The reason for the different results may be that the high-altitude environment increases the basal metabolic rate of the body, promotes protein breakdown, improves the body’s functional status, and prevents the generation of peripheral fatigue (Ma et al., 2023).

In this study, the stimulation site of tPCS was located in the frontal lobe, and the pulse characteristics of tPCS current can stimulate deep brain nuclei. Therefore, tPCS may regulate the stimulation of the frontal lobe from top to bottom, ultimately causing changes in testosterone and cortisol (Saavedra et al., 2014; Datta et al., 2013; Vasquez et al., 2017). Scholars have found in their studies that using transcranial direct current stimulation to the dorsolateral prefrontal cortex (DLPFC) or C3 (brain localization based on the 10–20 EEG system) can reduce cortisol concentration (Raimundo et al., 2012; Moreno-Duarte et al., 2014).

**FIGURE 6:**
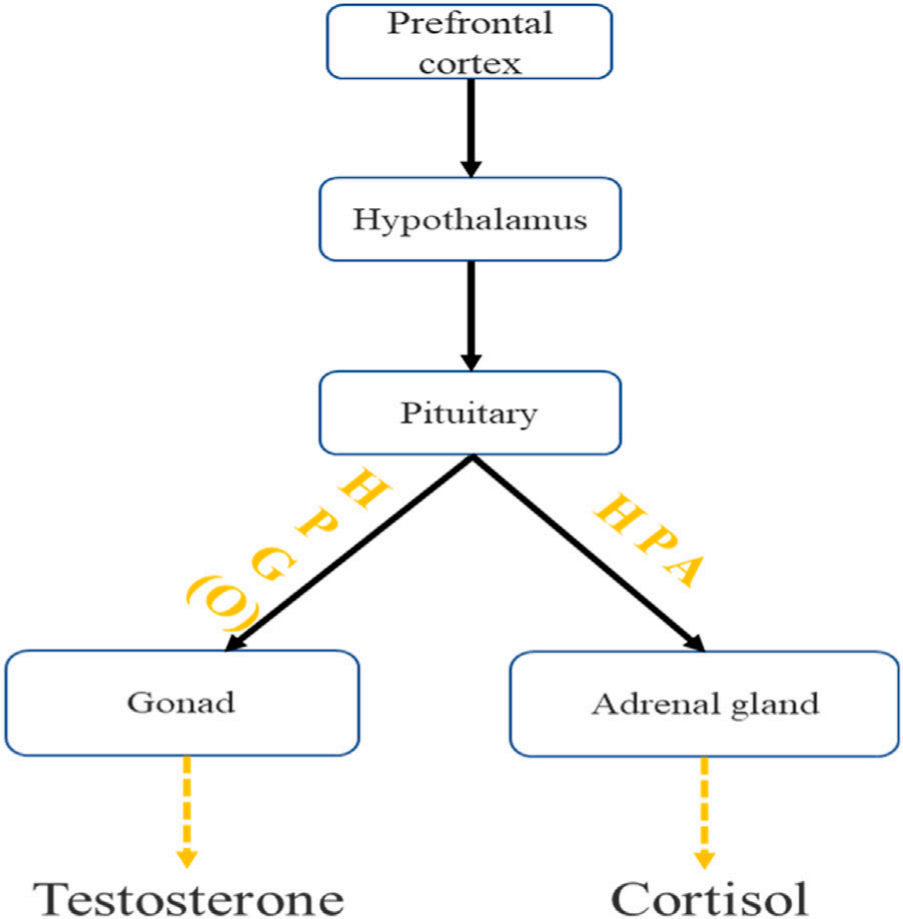
The pathway of action of tPCS on the central and peripheral nervous system.

Both tPCS and tDCS belong to transcranial electrical stimulation, with certain similarities in the target area and effect of stimulation, while the production of testosterone and cortisol is controlled by the hypothalamic-pituitary gonadal axis (HTPG) (Barbas et al., 2003), and there is a wide functional connection between the frontal lobe and the hypothalamus (Price et al., 2005). Therefore, We speculate that the pulse current of tPCS may affect the activity of HTPG through the frontal cortex (Shekhar et al., 2003; Shaffer et al., 2014), thereby promoting testosterone secretion, delaying the decrease in testosterone concentration, and ultimately maintaining individual motor behavior (Figure 6).

Moreover, the results of this study showed that the indexes T of peripheral fatigue in the two groups were highly correlated with the indexes Oxy-Hb and HbTot of central fatigue. This may suggest that there is an inherent connection between central fatigue and peripheral fatigue, but further physiological and biochemical experiments are needed to clarify their clear pathways of action.

### 4.3 The impact of fatigue on behavioral indicators

After exercise training, the behavioral indicators of both groups showed significant changes compared to before the tPCS intervention; Compared with the control group, the rate of change in the experimental group was lower, in which the difference of the change rate at reaction time was 18.92%, and the difference of the change rate at attention was 7.83%. This suggests that tPCS intervention after continuous moderate-intensity exercise can delay the rise in response time and the fall in target time after fatigue to some extent. Through correlation analysis, it was found that response was significantly correlated with both central fatigue and peripheral fatigue, while attention was not significantly correlated with both central fatigue and peripheral fatigue.

The reaction time indicates the speed of information processing in the central nervous system of the brain. When the central nervous system accelerates information transmission, the reaction time is shortened. Many scholars have found that the increase in cerebral perfusion will lead to an increase in central neuron activation, especially the increase of blood oxygen content can be reflected in the transmission speed of central nervous system signals, and the response time will be shortened when the central nervous system is improved (Lucas et al., 2012a; Lambrick et al., 2016; Lucas et al., 2012b) demonstrated this viewpoint through experiments that when cerebral blood flow in the frontal lobe increases, reaction time improves, which is consistent with the results of this study. This study found that after tPCS intervention, there was a significant negative correlation between the Oxy-Hb value and reaction time, the larger the Oxy-Hb value, the shorter the reaction time. Similar situations exist between attention and cerebral perfusion. Marshall et al. (2001) found in their study that after a decrease in cerebral blood flow, attention was impaired by 40%. However, in this study, no relationship was observed between target time and blood oxygen status. In fact, some scholars have proposed that both central fatigue and peripheral fatigue can affect reaction time and attention to a certain extent, but the degree of impact varies. Lin and Liu, (2014) found in long-term exercise-induced central fatigue that central fatigue prolongs reaction time; When peripheral fatigue occurs, it can reduce muscle strength reserves, inhibit the body’s athletic ability, and also affect reaction time (Skinner et al., 2005). Moreover, peripheral fatigue often occurs at the neuromuscular junction and muscle fibers (Ament and Verkerke, 2009; Maclaren et al., 1989), and the accumulation of fatigue metabolites can affect neuromuscular performance, leading to decreased motor performance (Chen et al., 2022). Neuromuscular fatigue can also affect motor control and proprioception, reducing the body’s decision-making ability and reaction time (Borotikar et al., 2008), Due to the involvement of nerves and muscles in both reaction time and attention, both central fatigue and peripheral fatigue can affect the motor behavioral characteristics of this study. This is inconsistent with the results of this study, possibly because the central nervous system involvement required to complete reaction time and attention behavior is greater than the involvement of peripheral muscles. In this experiment, the accumulation of peripheral fatigue occurred more frequently in the lower limb major muscle group during exercise, with a lower correlation with the upper limb minor muscle group completing these two types of sports behaviors; Therefore, the correlation between peripheral fatigue and sport behavior in this study is not significant.

Peripheral fatigue contributes relatively more to the decrease in muscle activity after short-term high-intensity exercise, while central fatigue contributes relatively more in long-term moderate-intensity exercise; The elimination of peripheral fatigue depends more on time, while the elimination of central fatigue is closely related to external intervention (Carroll et al., 2017). Central fatigue occurs in the central nervous system, usually due to obstacles in the transmission of neural signals. The main mode of regulation is neural regulation, which is characterized by accuracy and speed (Guo et al., 2023); Peripheral fatigue includes neuromuscular junctions, peripheral nerves, muscle cell membranes, calcium release mechanisms, and sliding filaments, mainly regulated by humoral regulation, which is characterized by broad and slow regulation (Ament and Verkerke, 2009; Wu et al., 2022). Therefore, tPCS improved the cerebral blood oxygen status and delayed the degree of central fatigue by directly intervening in the frontal lobe. However, the intervention of tPCS cannot directly affect peripheral biochemical indicators and must be regulated through neurohumoral regulation. Therefore, the effect of tPCS on central fatigue is greater than that on peripheral fatigue (Liu T. et al., 2016).

Regression analysis shows that the coefficient of determination of central fatigue on reaction time is over 80%, the R^2^ is 0.93 in the experimental group and 0.86 in the control group. This indicates that changes in reaction time are mainly influenced by central fatigue, and after tPCS intervention, the impact of central fatigue on reaction time further increases.

The research of Liu Jianxiu et al. (2016) supports the results of this experiment. He believes that reaction time can be used as a behavioral indicator to evaluate central fatigue. Reaction time mainly reflects the flexibility of brain neural activity. When fatigue occurs, phenomena such as decreased brain flexibility, limited individual activity level, and delayed response may occur.

The central nervous system is sensitive to factors such as arterial oxygen partial pressure, arterial oxygen content, and arterial oxygen saturation, which together affect brain function (Calbet et al., 2003). In this study, tPCS is stimulated in the frontal lobe of the brain, which has a great effect on the central nervous system. Therefore, the impact of tPCS on response time is mainly achieved by changing the central fatigue state. Rasmussen et al. (2007) studied the effect of brain oxygenation on motor behavior activation through the contraction of finger static maximum isometric autonomous grip strength, they found that the decrease in finger grip level may not only be caused by peripheral neuromuscular factors but also by the imbalance of brain blood oxygen transport and demand caused by continuous exercise. When the brain’s blood oxygen concentration decreases, it inhibits the activity of efferent neurons, ultimately, the individual’s motor behavior performance was reduced, and the intervention of tPCS activated neurons, so the sport behavior can be improved. Kataoka et al. (2022) studied from the perspective of exercise programs and proposed that peripheral fatigue contributed more to the reduction of muscle contraction during short-duration, high-intensity exercise, while central fatigue contributed more to the reduction of muscle contraction during medium-intensity exercise with a longer duration. In this study, the subjects engaged in long-term moderate-intensity exercise, so we believe that under the intervention of tPCS, the changes in reaction time are mainly influenced by central fatigue. The study by Goodall et al. (2010) also showed that central fatigue caused by cerebral oxygenation can inhibit motor drive in the corticospinal cord, leading to changes in sports behavior. This provides a potential logic that central fatigue can affect sports behavior, which is consistent with the results of this study.

## 5 Limitations of research

1 This study lacks a clear mathematical relationship between central fatigue and peripheral fatigue, and cannot analyze the intrinsic relationship between central fatigue and peripheral fatigue in more detail. In fact, due to the widespread presence of the nervous system in the central and peripheral regions, central fatigue and peripheral fatigue have their own characteristics, but there is also a certain degree of intersection. However, distinguishing this independent yet unified relationship is a worthwhile and challenging task.
2 This study did not link sports or tasks with central and peripheral fatigue, so the application of tPCS in specific competitions is not clear enough and lacks detailed application guidelines.

## 6 Conclusion

1 tPCS intervention can delay the development of central fatigue and peripheral fatigue.
2 tPCS intervention has a greater effect on central fatigue than peripheral fatigue.
3 The effect of tPCS intervention on response time was mainly achieved by changing the central fatigue state.

## Data Availability

The raw data supporting the conclusions of this article will be made available by the authors, without undue reservation.
